# Attitudes to Climate Change Risk Among Older People: New Evidence From the English Longitudinal Study of Ageing

**DOI:** 10.1093/geroni/igaf122.1264

**Published:** 2025-12-31

**Authors:** Giorgio Di Gessa, Paola Zaninotto

**Affiliations:** University College London, London, England, United Kingdom; University College London, London, England, United Kingdom

## Abstract

Public perceptions of climate change’s risks are essential to ensure support for the strategies necessary to achieve carbon emissions goals. Although older people are often considered most vulnerable to the effects of climate change and extreme weather, their perspectives have often been overlooked. Therefore, in this study, we aim to describe public attitudes towards climate change risk among older people in England, using data from the latest wave of the English Longitudinal Study of Ageing (2023/24) that assessed, for the first time, attitudes towards climate change risk. We applied latent class analysis to categorise respondents according to their level of agreement with six statements on climate change risks. Multinomial regression modelling was then used to investigate the extent to which demographic, socioeconomic, health, and social engagement were associated with different classes of perceptions. In our preliminary analysis, we identified four distinct attitude classes: the “Sceptical” (6%), the “Very Worried” (33%), the “Slightly Concerned” (40%), and the “Unsure” (21%). Results suggest that people aged 70 and older and men were more likely to be sceptical or unsure about climate change than those in their 50s and women. Also, wealthier and highly educated older people were more likely to be worried, with respondents actively participating in political and religious lives being the most likely to be worried about climate change. These initial findings suggest that public information campaigns regarding climate change risk are still necessary and should be tailored specifically to those older people who remain sceptical or unaware of these issues.

